# Investigating causality with fecal microbiota transplantation in rodents: applications, recommendations and pitfalls

**DOI:** 10.1080/19490976.2021.1941711

**Published:** 2021-07-30

**Authors:** Cassandra E. Gheorghe, Nathaniel L. Ritz, Jason A. Martin, Hannah R. Wardill, John F. Cryan, Gerard Clarke

**Affiliations:** a Department of Psychiatry and Neurobehavioral Science, University College Cork, Cork, Ireland; b Department of Anatomy and Neuroscience, University College Cork, Cork, Ireland; c APC Microbiome Ireland, University College Cork, Cork, Ireland; d Precision Medicine, South Australian Health and Medical Research Institute (SAHMRI), Adelaide, Australia; e Adelaide Medical School, the University of Adelaide, Adelaide, Australia; f INFANT Research Centre, University College Cork, Cork, Ireland

**Keywords:** Fecal microbiota transplantation, rodent, human, germ-free, antibiotics, gnotobiotic, microbiota depletion, methods, experimental design

## Abstract

In recent years, studies investigating the role of the gut microbiota in health and diseases have increased enormously – making it essential to deepen and question the research methodology employed. Fecal microbiota transplantation (FMT) in rodent studies (either from human or animal donors) allows us to better understand the causal role of the intestinal microbiota across multiple fields. However, this technique lacks standardization and requires careful experimental design in order to obtain optimal results. By comparing several studies in which rodents are the final recipients of FMT, we summarize the common practices employed. In this review, we document the limitations of this method and highlight different parameters to be considered while designing FMT Studies. Standardizing this method is challenging, as it differs according to the research topic, but avoiding common pitfalls is feasible. Several methodological questions remain unanswered to this day and we offer a discussion on issues to be explored in future studies.

## Introduction

**Figure 1.**
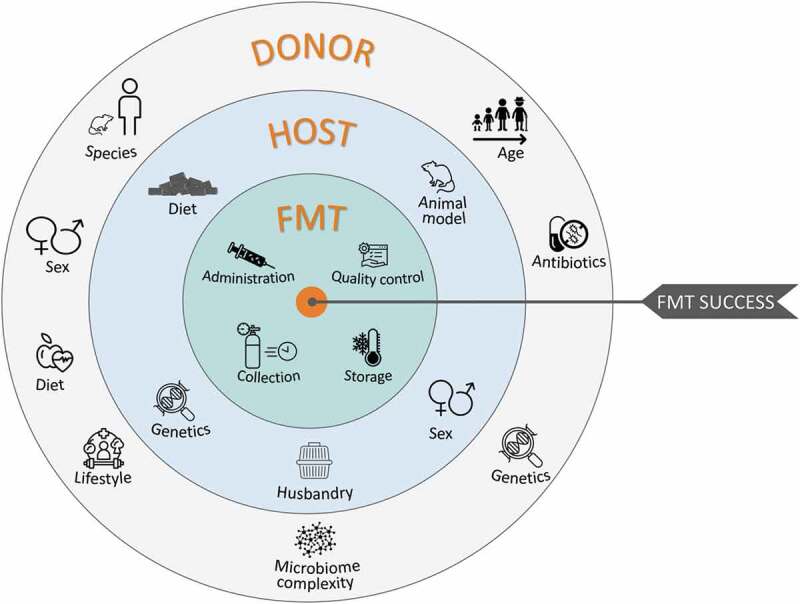
Parameters influencing the experimental design of fecal microbiota transplant studies

The human gastrointestinal (GI) tract houses a dynamic population of microorganisms, known as the gut microbiota, which have a major impact on the host during homeostasis and disease.^1^ By comparing conventional rodents with germ free ones (i.e. devoid of all microorganisms), an important role of the intestinal microbiota is attributed to the symptoms expressed and prominent features of numerous diseases.^2^ The information garnered from this approach has been supplemented with supporting evidence from a number of complementary experimental strategies. However, despite the invaluable information gathered from preclinical studies, in the majority of cases, causality remains unclear. Recently, we have begun to understand how host physiology and behavior – in rodents – can be affected solely by the transplantation of fecal microbiota from a donor (human, rodent, etc.). This method has become common for assessing causality arising from the complex host-microbiota interactions, as it is a powerful way to understand the strong involvement of intestinal microorganisms on our overall health. Indeed, fecal microbiota transplantation (FMT) studies have implicated the gut microbiota in gastrointestinal disorders like irritable bowel syndrome (IBS)^3^ and metabolic disorders such as Type 2 Diabetes and Obesity.^4^ Even more surprisingly, FMT studies highlighted the potential involvement of the intestinal microbiota in CNS diseases including: neurodegenerative disorders such as Parkinson’s disease,^5^ multiple sclerosis,^6^ Alzheimer’s disease;^7^ developmental disorders like autism spectrum disorder^8^ but also some psychiatric conditions including schizophrenia^9^ and depression.^10^ FMT involves many steps and requires careful experimental design. Indeed, laboratories using this method to study different topics adapt the experimental parameters to meet the requirements of their own research topic and experimental readouts. FMT studies are the result of a number of important experimental design choices: human or rodent donors, rodent strain and sex, recipient animal models (e.g.: germ-free (GF), antibiotics- or laxative-treated, conventional animal etc.), factors influencing the recipient animals (feeding, housing conditions), fecal slurry preparation (processing, storage, concentration, administration method) and the method used to ascertain the degree and stability of engraftment (see Figure 1). The lack of explicit explanations around the rationale for the choices made in the selection of these parameters is reflected in a great deal of variation in experimental protocols that often complicates the interpretation of results and makes inter-study comparison difficult. In addition, the constant emergence of new information regarding the best way to approach a specific parameter of the FMT process, makes it challenging to plan best practice during FMT. For example, new information has recently become available on the establishment of microbiota in gnotobiotic animals.^11^ Careful consideration of these factors is critical to obtain reliable and robust results. While FMT has undoubtedly led to improvements in our understanding of health and disease, this rapidly evolving scientific approach is undermined by the lack of standardization in its methods, application, and interpretation. As such, the intention of this review is to equip basic, clinical and translational scientists with a clear understanding of the principles of the FMT technique in preclinical research with the aim to increase the quality of research outcomes and their translational relevance. This review focuses only on rodent recipients as this is the most widely used model in pre-clinical research to assess causality of the intestinal microbiota. By identifying key elements in FMT methodology that are subject to variable implementation, we hope to provide recommendations based on the state of the art in this field and highlight the promise and pitfalls of this approach as it is increasingly used in attempts to define a potential causal role of the gut microbiota in various aspects of health and disease. Our goal is to provide scientists wishing to use this technique in rodent research models with an overview of what has been done so far, the problems encountered and the best way to approach this technique according to the questions under evaluation. We believe that although it may be appropriate for studies to adopt bespoke protocols according to the topic being studied, certain principles and parameters of the experimental design can be further considered and standardized to best optimize the reliability of this method in each particular context. Clinical guidelines for the use of FMT in the treatment of disorders (e.g. *C. difficile* infection) are outside the scope of this review. However, relevant clinical findings are included where appropriate as some factors are given more consideration in the clinical literature than preclinical studies.

### Animal models

**Figure 2.**
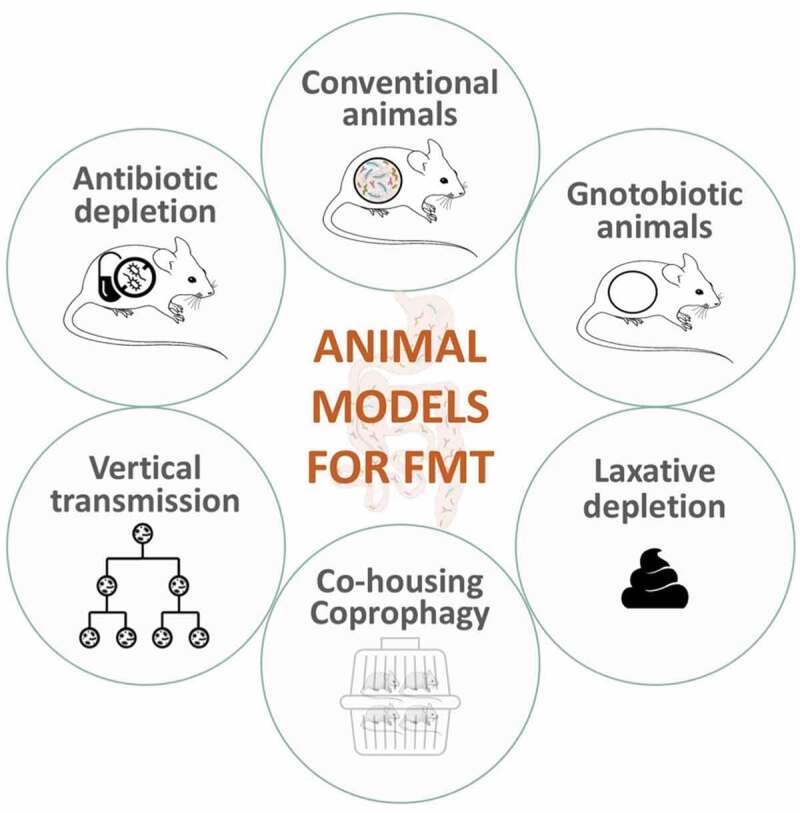
Most commonly used animal models as recipients for fecal microbiota transplants

Several animal models are used in FMT studies, each with advantages and limitations (see Figure 2). Researchers need to carefully consider the goals of their studies to determine the most suitable approach. (See Table 1 and 3)

**Table 1.** Frequently used antibiotics for gut microbiota depletion and their characteristics

Antibiotics	Spectrum of activity	Intestinal Absorption
Ampicillin (± sulbactam)	Broad spectrum (mostly Gram +)	Extensive 30–55%
Ciprofloxacin	Broad spectrum	Extensive
Streptomycin	Broad spectrum	Poor
Neomycin	Broad spectrum	Poor
Kanamycin	Broad spectrum	Unknown
Imipenem (Cilastatin)	Broad spectrum: both aerobic and anaerobic bacteria	Poor
Vancomycin	Gram-positive Bacteria	Poorly absorbed from GI, systemic absorption (up to 60%) may occur following intraperitoneal administration
Metronidazole	Narrow spectrum: anaerobic bacteria	Complete: lipophilic , rapidly and widely distributed after absorption.
Gentamicin	Mostly Gram negative	Poor^30^
Colistin	Gram negative Bacilli	Very poor absorption from gastrointestinal tract

**Table 2.** Advantages and limitations: administration of antibiotics

	Advantages	Limitations
Drinking water (Table 4)	Suitable for repeated administration Least stressful method Good absorption in GI tract	Single housing necessary if the dose needs to be monitored Consumption variability if grouped together Unpalatability can restrict intake leading to dehydration^26^
Oral gavage (Table 5)	Dosage monitored Good absorption in GI tract	Not very suitable for repeated administration The gavage procedure is stressful for the animal Repeat dosing should be carried out at approx. the same time each day Severe adverse effect if gavage tubes are incorrectly placed Technically difficult method
Combination (Table 6)	Abx can be administered accordingly to their pharmacokinetics Good absorption in GI tract

**Table 3.** Comparisons of different animal models for FMT

	Advantages	Limitations
**Gnotobiotic animals** GF	Microbial depletion guaranteed (within detection limits) No competition with the commensal microbiota to colonize the gut Best for testing specific mechanisms of interventions on host	No similar condition in humans to which it can be compared Experimental groups to which we can compare recolonized GF animals to: conventional animals, GF animals, or re-colonized animals? Expensive, requires access to specialized equipment and training Altered development (immune system very impacted)
**Gnotobiotic animals** -Defined microbiota (SPF)	Absence of specific pathogen	Basal composition unknown Competition with commensal microbiota to colonize the gut
**Gnotobiotic animals** -Defined microbiota (ASF)	Simplified model of a commensal microbiota	Competition with commensal microbiota to colonize the gut Not representative of a real microbiome-host interaction
**Antibiotic-treated animals**	Born and raised with a conventional microbiota Allow us to study specific life stages	Overgrowth of pathogenic species possible Antibiotic resistance genes Systemic side effects Antibiotic treatment varies across studies
**Conventional animals**	No abnormal development or side effects of a treatment	Unknown basal composition Native gut microbiota exert different selective pressures on exogenous colonizers
**Laxative-depletion**	Comparable to what is currently done in human Depletion seems to be effective at certain doses	Side effects of laxative use are not well-known Paucity of studies using this model in pre-clinical settings
**Vertical microbiota transmission model**	Natural transmission of the gut microbiota No abnormal development or side effects of a treatment	Not enough information on vertically or horizontally transmissible strains
**Bedding material/coprophagy**	Alters cecal content but also metabolic features and cognition Frequency of coprophagy can be beneficial (not a one-time screenshot of the donor’s microbiota) Easiest and least invasive/stressful model of transferring microbes	Not suitable for human to mice FMT Dose and frequency of transfer is not controlled or guaranteed Could imply single housing of rodents to control for coprophagy Bacterial transmission not targeted and probably restricted to oxygen-friendly microorganism

**Table 4.** Drinking water – protocols for antibiotic-induced gut depletion

DURA-TION	ANTIBIOTIC COCKTAILS CONCENTRATIONS	DISEASES (ICD 10)	PUBLICA-TIONS
2 days	streptomycin (500 g/ L)	Diseases of the circulatory system (Stroke)	^32^
	ciprofloxacin (0.2 g/L) + metronidazole (1 g/L)	Endocrine, nutritional and metabolic diseases (Obesity)	^33^
5 days	ampicillin (0.5 g/L)	Infectious disease	^34^
1 week	ampicillin (1 g/L)	GI disorder (due to antibiotic- and chemotherapy-induced gut dysbiosis)	^35^
1 or 2 weeks	amoxicillin–clavulanic acid (1 g/L)	Diseases of the circulatory system (Stroke)	^36^
7 days	S**ystemic antibiotic cocktail**: ampicillin (1 g/L) + cefoperazone sodium salt (1 g/L) + clindamycin (1 g/L) OR **Non-absorbable antibiotic cocktail**: ertapenem sodium (1 g/L) + neomycin sulfate (1 g/L) + vancomycin hydrochloride (1 g/L)	FMT protocol	^37^
7 days	ampicillin (0.01 g/1 L) + metronidazol (0.01 g/L) + neomycin (0.01 g/L)	Diseases of the digestive system (Hepatic steatosis)	^38^
10 days	vancomycin (1 g/L) + metronidazole (1 g/L) + polymyxin B (1 g/L) + cefotaxime (2 g/L)	FMT protocol	^39^
	cefoxitin (1 g/L) + gentamicin (1 g/L) + metronidazole (1 g/L) + vancomycin (1 g/L)	Immune system	^40^
10–14 days	ampicillin (1 g/L) + metronidazole (1 g/L) + neomycin sulfate (1 g/L) + vancomycin (0.5 g/L)	Infectious disease	^41^
14 days	ampicillin (1 g/L) + metronidazole (1 g/L) + neomycin (1 g/L) + vancomycin (0.5 g/L)	Immune system	^42^
14 days	ampicillin (1 g/L) + metronidazole (1 g/L) + neomycin sulfate (1 g/L)	Diseases of the nervous system (Alzheimer's disease)	^43^
14 days	ampicillin (1 g/L) + metronidazole (1 g/L) + neomycin (1 g/L) + vancomycin (0.5 g/L) supplemented with 1% (wt/vol) glucose.	Immune system	^44^
14 days	ampicillin (1 g/L) + metronidazole (1 g/L) + neomycin (1 g/L) + vancomycin (0.5 g/L)	Endocrine, nutritional and metabolic diseases (Obesity)	^45^
14 days	ampicillin (1 g/L) + metronidazole (1 g/L) + neomycin sulfate (1 g/L)	Mental and behavioral disorders (Depression)	^46^
14 days	ampicillin (1 g/L) + vancomycin (0.5 g/L) + neomycin (1 g/L) + metronidazole (1 g/L)	Antibiotic effects	^47^
2–3 weeks	ampicillin (1 g/l) + streptomycin (5 g/l) + colistin (1 mg/ml) or vancomycin alone (0.25 g/L) or imipenem alone (0.25 g/L) or colistin alone (2.103 U/ml)	Neoplasms (Cancer)	^48^
2–3 weeks	ampicillin (1 g/L) + vancomycin (0.5 g/L) + neomycin (1 g/L) + metronidazole (1 g/L)	Infectious diseases	^49^
3 weeks	ampicillin (1 g/L) + neomycin (1 g/L) + streptomycin (1 g/L) + kanamycin (1 g/L) and/or anti-fungal cocktail drinking water amphotericin (0.2 g/L) + fluconazole (0.5 g/L) + 5-fluorocytosine (0.5 g/L)	Infectious diseases	^50^
3 weeks	ampicillin (1 g/L) + metronidazole (1 g/L) + neomycin sulfate (1 g/L) + vancomycin (0.5 g/L)	Infectious diseases	^51^
3 weeks	vancomycin (0.5 g/l) + ampicillin (1 g/l) + kanamycin (1 g/l) + metronidazole(1 g/l)	Immune system	^52^
3 weeks	ampicillin (1 g/L) + metronidazole (1 g/L) + neomycin (1 g/L) + vancomycin (0.5 g/L)	Immune system	^53^
3 weeks	vancomycin (0.5 g/L) + neomycin sulfate (1 g/L)+ ampicillin (1 g/L) + metronidazole (1 g/L)	Immune system	^54^
3 weeks	ampicillin (1 g/L) + metronidazole (1 g/L) + vancomycin (0.5 g/L) + neomycin trisulfate (1 g/L)	Diseases of the digestive system	^55^
4 weeks	ampicillin (1 g/L) + vancomycin (0.5 g/L) + polymyxin (0.1 g/L)	Immune system	^56^
4 weeks	ampicillin (1 g/L) + neomycin (1 g/L) + metronidazole (1 g/L) + vancomycin (0.5 g/L)	Diseases of the musculoskeletal system and connective tissue	^57^
4 weeks	vancomycin (0.5 g/L) + neomycin (1 g/L) + ampicillin (1 g/L) + metronidazole (1 g/L)	Diseases of the digestive system	^58^
4 weeks	ampicillin (1 g/L) + neomycin (1 g/L) + metronidazole (1 g/L) + vancomycin (0.5 g/L) or vancomycin (0.5 g/L) alone	Bone formation	^59^
4–5 weeks	ampicillin (1 g/L) + neomycin (0.5 g/L) + streptomycin (0.5 g/L) + vancomycin (0.5 g/L)	Diseases of the blood and blood-forming organs and certain disorders involving the immune mechanism	^60^
7 weeks	ampicillin (1 g/L) + vancomycin (0.5 g/L) + ciprofloxacin (0.2 g/L) + imipenem plus cilastatin (250 mg/L) + metronidazole (1 g/L)	Mental and behavioral disorders (depression)	^61^

**Table 5.** Oral gavage – protocols for antibiotic-induced gut depletion

DURATION	ANTIBIOTIC COCKTAILS CONCENTRATIONS	DISEASES (ICD 10)	PUBLICATIONS
3 days	ampicillin (1 g/L) + neomycin (0.5 g/L) + vancomycin (0.5 g/L) + metronidazole (1 g/L)	Aging	^62^
3 days	ampicillin (500 mg) + vancomycin (250 mg) + neomycin (500 mg) + metronidazole (250 mg)	Diseases of the digestive system	^63^
3 days	ampicillin (1 g/l) + streptomycin (5 g/L) + colistin (1 g/L) + vancomycin (0.25 g/L)	Neoplasms (Cancer)	^64^
5 days	ciprofloxacin (0.1 g/L) + ampicillin (0.5 g/L)	Diseases of the digestive system	^65^
5 days	ampicillin (200 mg/kg) + neomycin sulfate (200 mg/kg) + metronidazole (200 mg/kg) + vancomycin (100 mg/kg)	Infectious diseases	^66^
7 days	ampicillin (1 g/ml) + metronidazole (1 g/ml) + neomycin sulfate (1 g/ml) + vancomycin (0.5 g/ml)	Diseases of the nervous system (Encephalomyelitis)	^67^
7 days	ampicillin (200 mg/kg) + neomycin (200 mg/kg) + metronidazole (200 mg/kg) + vancomycin (100 mg/kg)	FMT protocol	^24^
10 days	metronidazole (0.1 mg/g bodyweight) + ampicillin (0.26 mg/g bodyweight) + neomycin (0.26 mg/g bodyweight) + vancomycin (0.13 mg/g bodyweight)	Disease of the circulatory system	^68^
11 days	ampicillin (43.2 mg) + bacitracin (108.0 mg) + meropenem (21.6 mg) + neomycin (108.0 mg) + vancomycin (6.48 mg) in 4,5 mL of distilled water	Antibiotic effects	^31^
14 days	ampicillin (1 g/L) + neomycin sulfate (1 g/L) + metronidazole (1 g/L)	Mental and behavioral disorders (depression)	^69^
14 days	ampicillin (1 g/L) + neomycin sulfate (1 g/L) + metronidazole (1 g/L)	Mental and behavioral disorders (depression)	^70^
14 days	ampicillin (0.2 g/L) + neomycin (0.2 g/L) + metronidazole (0.2 g/L) + vancomycin (0.1 g/L)	Neoplasms (Cancer)	^71^
14 days	ampicillin (1 g/L) + vancomycin (0.5 g/L) + neomycin (1 g/L) + metronidazole (1 g/L) + ciprofloxacin (1 g/L) in some experiments	Diseases of the blood and blood-forming organs and certain disorders involving the immune mechanism	^47^
14 days	ampicillin (0.25 mg/day) + gentamicin (0.25 mg/day) + metronidazole (0.25 mg/day) + neomycin (0.25 mg/day) + vancomycin (0.125 mg/day)	Mental and behavioral disorders (depression)	^72^
14 days	ampicillin (2.5 g/L) + metronidazole (2.5 g/L) + neomycin (2.5 g/L) + vancomycin (1.0 g/L)	Sleep	^73^
3 weeks	ampicillin (1 g/L) + metronidazole (1 g/L) + neomycin (1 g/L) + vancomycin (0.5 g/L)	Immune system	^74^

**Table 6.** Combined oral gavage and drinking water administration – protocols for antibiotic-induced gut depletion

DURATION	ANTIBIOTIC COCKTAILS CONCENTRATIONS	DISEASES (ICD 10)	PUBLICATIONS
5 consecutive days of gavage + 5 weeks in drinking water	**Oral gavage**: ampicillin, neomycin, metronidazole and vancomycin for 5 days (0.2 mL: 10 mg of each antibiotic per mouse per day) **Drinking water**: ampicillin (1 g/L) + neomycin (1 g/L) + metronidazole: (1 g/L) + vancomycin: (0.5 g/L) for 5 weeks	Endocrine, nutritional and metabolic diseases (Obesity)	^75^
7 days	**Oral gavage**: vancomycin (50 mg/kg) + neomycin (100 mg/kg) + metronidazole (100 mg/kg); **Drinking water**: Ampicillin (1 g/L)	Disease of the circulatory system (Stoke, Seizure)	^76^
14 days	**Oral gavage**: vancomycin (5 g/L) + neomycin, (10 g/L) + metronidazole (10 g/L) + amphotericinB (0.1 g/L); **Drinking water**: ampicillin (1 g/l) + last 3 days: omeprazole via oral gavage (50 mg/kg, once a day)	Disease of the nervous system (Epilepsy)	^77^
14 days	**Oral gavage**: 3 days of amphotericin-B (1 mg/kg BW) every 12 h + from day 3 **Drinking water**: ampicillin (1 g/L) + **Oral gavage**: every 12 h vancomycin (50 mg/kg BW) + neomycin (100 mg/kg BW) + metronidazole (100 mg/kg BW) + + amphotericin-B (1 mg/kg BW)	Antibiotic-depletion protocol	^26^
14 days	**Oral gavage**: Vancomycin (250 mg) + neomycin-sulfate (500 mg) + ampicillin (500 mg) + metronidazole (500 mg) supplemented with 10 g grape Kool-Aid in 500 mL water + **Driking water**: Ampicillin (1 g/L)	Disease of the respiratory system (Influenza)	^78^
17 days	**Oral gavage**: 3 days of amphotericin-B (1 mg/kg BW) every 12 h + from day 3 **Drinking water**: ampicillin ad libitum (1 g/L) + **Oral gavage**: every 12 h vancomycin (50 mg/kg BW) + neomycin (100 mg/kg BW) + metronidazol (100 mg/kg BW) + + amphotericin-B (1 mg/kg BW)	FMT Protocol	^79^

### Gnotobiotic animals

Gnotobiotic animals are used as a resource to understand the role of the intestinal microbiota in relevant conditions. Starting a GF line – rodents devoid of all microorganisms – requires aseptic removal of a pup from the mother to avoid any exposure to its mother’s microorganisms. The animals are reared in sterile cages and reproduce under aseptic conditions.^2^ The term “gnotobiotic animals” includes GF animals but also GF animals recolonized with a defined microbiota to study specific germs (mono-associated or defined microbiota animals).^12^ Gnotobiotic rodents are fundamental tools to understand the role of the gut microbiota and have been employed in a number of studies to successfully demonstrate the adoptive transfer of various behavioral phenotypes.

- GF rodents are raised in isolators and are devoid of all microorganisms including: bacteria, viruses, fungi, archaea, protozoa and other parasites – within the range of detection limits used (sequencing- or cultured-based techniques). The use of this animal model has contributed greatly to the microbiome field. It is an excellent model for understanding the extent to which the intestinal microbiota impacts on health and overall physiology. Therefore, it appears as a reliable recipient for FMT studies. The most beneficial aspect of using this model to receive an exogenous intestinal microbiota is the lack of competition with resident microbes to colonize the gut. However, it is also important to note that a GF state leads to abnormal development in many features of interest: immune system development,^13^ enteric nervous development,^2^ neurodevelopmental deficits^2^ and altered behavioral responses.^2^ This renders interpretation challenging as it has been shown that the introduction of a complex microbiota will sometimes normalize those aspects of the host physiology that are compromised by growing up GF. Based on this knowledge, it is advisable not to rely solely on comparisons between GF animals and ex-GF animals. For example, the use of both conventionally raised and ex-GF animals (i.e. born and delivered under GF conditions and subsequently removed from isolators for colonization) as control groups, when comparing to GF animals, can help parse a role for the gut microbiota in the abnormal development of germ-free mice vs an ongoing contribution during adulthood. Moreover, standardized behavioral tests can require some adjustments for germ-free rodents – as they exhibit abnormal behavior – to avoid floor and ceiling effects. For instance, GF mice exhibit an exaggerated HPA-axis response. Therefore, understanding the effect of a fecal transplant, where it is anticipated to activate the stress-response system on their response to a restraint stress paradigm, will be more challenging.^14^ Additionally, the cost of breeding and handling animals under sterile conditions is significant.^15^ Detailed protocols for maintaining and generating GF animals are available^16^ but the requirement for a dedicated facility and expertise means access is not routine in many institutions. To counteract the expenses of isolators, a cost-effective solution would be the use of individually ventilated cages to house GF mice according to Lange et al. protocol.^12^ They present a protocol for the use of a conventional IVC system operating under containment mode and provide details for cage changing and sampling without compromising the sterile status. Importantly, the use of IVC system might have an impact for behavioral studies.^17^

(ii) Defined microbiota animals are generated by colonizing GF animals with one or more specific microorganisms. Specific Pathogen Free (SPF) rodents,^18^ widely used in FMT studies, are defined as animals free from specific pathogens but otherwise having an undefined microbiota. Differences exist between breeding establishments with regard to the list of excluded pathogens in SPF mice used, therefore not allowing for proper standardization of SPF animals.^18^ With the hope of standardization between studies, Schaedler et al.^19^ proposed the “Altered Schaedler Flora (ASF)” rodent model, representing a simpler model of commensal microorganisms composed of only eight bacterial species. (Methodology papers regarding this model can be found in Dewhirst et al. and Biggs et al.^20^ review). ASF rodent use has evolved since 1965 toward a controlled and simplified model of a more complex microbiota to study microbiome-host interactions.^21^ However, in some studies, this low diversity of microorganism can result in intermediate phenotypes between GF and SPF.^22^ This model is limited in its ability to fully predict the impact of the intestinal microbiota on its host since it is only a simplified representation of the gut microbiota.

In conclusion, GF rodents are an insightful ‘knock-out’ model that have helped to highlight the importance of a rich and diverse microbiota for health and diseases. Rodents colonized with a simplified microbiota are useful for standardizing research between groups and provide a simplified model for studying microbiome-host interaction, thereby reducing the tremendous variability that exists when studying conventionally raised rodents. It also reduced developmental abnormalities seen in GF animals. One study raises important concerns on the use of SPF mice for translational studies in the immunology field, given that this could compromise the establishment of a fully mature adult-like immune system.^23^ Limitations of the use of gnotobiotic animals led to the proliferation of alternative microbiota-depletion approaches.

### Antibiotic-treated animals

The antibiotic-depleted microbiota model is an alternative to gnotobiotic rodents widely used for a number of reasons: it is simpler in terms of experimental design and less expensive, it does not require access to specialized housing equipment, and it circumvents the limitations of a GF animal model. Antibiotics are a simple and accessible way to induce gut microbiota depletion – even though it never fully removes all microorganisms. It has been suggested that the use of juvenile mice subjected to initial microbiota depletion constitutes a valid alternative to GF mice in microbiota transfer studies.^24^ ‘Pseudo-depletion’ of gut microbes can be adjusted depending on: the type of antibiotics used (bactericidal or bacteriostatic) or targeted microbes (gram-negative or positive bacteria, narrow- or broad-spectrum antibiotics). When targeting specific types of bacteria, it is important to consider how it impacts the overall ecological network and might cause the multiplication of some strains at the expense of others. For an effective depletion of gut bacteria, an antibiotic cocktail containing several antibiotics that together give a broad spectrum of activity is necessary: after 2 weeks of broad-spectrum antibiotic administration, 72% to 86% reduction in bacterial load was reported.^25,26^ One study compared the depletion of gut microbes by administering a single broad-spectrum antibiotic (Ampicillin, Doxycycline or Ciprofloxacin) and concluded that each option individually elicited unique taxonomic changes^27^ which reinforces the view that an antibiotic cocktail would be more appropriate than a single broad-spectrum antibiotic. Another study compared 16S copies number in fecal pellets after 2 weeks of administration of either: an antibiotic cocktail (1 g/L ampicillin, 1 g/L metronidazole, 1 g/L neomycin, 0.5 g/L vancomycin), a single antibiotic (1 g/L ciprofloxacin) or a combination of antibiotics (1 g/L neomycin and 1 g/L metronidazole; 0.5 g/L vancomycin and 1 g/L ampicillin). Only the antibiotic cocktail and the combination vancomycin/ampicillin were significantly different than vehicle-treated animals.^28^ The normal commensal organisms of rodents are mainly gram-positive bacteria.^29^ Hence, the use of limited-spectrum antibiotics that target only gram-positive bacteria, for example, may lead to the proliferation of gram-negative bacteria.^29^ The antibiotic cocktail can be supplemented with antifungals (amphotericin B, pimaricin, natamycin) to prevent fungal proliferation during antibiotic treatment. Table 1. offers an overview of frequently used antibiotics for gut microbiota depletion.

#### Administration

The antibiotic cocktail – drugs and dosage – to be used for a significant standardized knockdown of gut bacteria still needs to be determined: this incomplete and variable depletion of the microbiome is currently a biological limitation of this approach. However, it may be advanageous that antibiotics used for depletion studies are non-absorbable by the gut (see Table 1). A short-term oral gavage treatment with non-absorbable antibiotics has been proposed (ampicillin, bacitracin, meropenem, neomycin, vancomycin) – except for ampicillin which was able to reach the systemic circulation but was undetectable in the brain.^31^ The dose, frequency and cocktail of antibiotics must be considered to induce a depletion of the intestinal microbiota that could approach a germ-free state, although there are a lack of studies specifically examining the impact of all the different antibiotic cocktails that have been used to date (see Table 1 for some estimates of depletion). Tables 4, 5 and 6 offers a summary of doses/frequency, concentration and antibiotic cocktails used in the literature so far. In general, it appears that 7 days of antibiotic administration is sufficient to ensure a severe microbiota depletion.^37^ This is likely a reasonable time to avoid antibiotic resistance and overgrowth of pathogenic bacteria. If the treatment is short enough (e.g. 5–7 days) and followed by FMT, the side effects of the antibiotics can be reversed. Unpublished data from our laboratory suggests that substantial knockdown can happen even after 24 hours of an antibiotic cocktail (Vancomycin, Imipenem, Gentamicin, Ampicillin) in mice.

Antibiotics can be administered for gut bacteria depletion via several routes: drinking water, oral gavage or a combination of both and intraperitoneal injection. By comparing them, it was observed that oral treatment – by drinking water or oral gavage – but not intraperitoneal injection treatment substantially reduced gut commensal microbiota.^67^ Administration of antibiotics in drinking water is very common as it is easy and noninvasive for the animal. However, *ad libitum* administration prevents control over the rate of consumption and consideration should be given to the stability of the antibiotics in water. It is recommended to record daily water intake to get an estimate of their antibiotic consumption, even though a number of rodents are usually caged together.

Oral gavage is a more reliable method for controlling the frequency and dose of antibiotics administered but is not ideal for long periods of antibiotic administration. This technique requires practice to minimize adverse events associated with it^80^ such as habituation to handling prior to the study.^80^ If large volumes are required, a slow delivery rate is recommended.^80^ Without habituation, rodents can show signs of stress as long as 1 h after gavage.^81^ Oral gavage with sucrose-precoated gavage needles can measurably decreases signs of stress thereby improving animal welfare during this procedure;^82^ even though sucrose consumption might be a confounding factor in some studies. A comparison of mice receiving: a single-course systemic antibiotic cocktail, a non-absorbable antibiotic cocktail and a three-course antibiotic cocktail altering systemic and non-absorbable antibiotics has shown that multiple courses of alternating antibiotic cocktails allowed sustained engraftment of human gut microbiota – numerically comparable to the colonization of GF mice.^37^ However, for the same human donor, the gut microbiota composition of GF or antibiotic-treated mice recipients differed significantly. This implies that regardless of the model chosen, the engraftment dynamics will be different since they have different gastrointestinal environment. This information is also important when interpreting the results. It is possible to combine administration by drinking water and oral gavage, allowing administration according to the pharmacokinetics of the antibiotics. Table 2 summarizes advantages and limitations of each based on Turner et al.^81^ and Morton et al.^83^ work.

#### Use of antibiotics: important considerations

Some important aspects need to be considered when designing an antibiotic depletion experiment. Antibiotic consumption may produce temporary side effects or in some cases permanent ones – especially at critical developmental stages^1^. Side effects of antibiotic-induced depletion are not yet fully understood. For example, some antibiotics can alter metabolic homeostasis (ampicillin, vancomycin, metronidazole, neomycin),^84^ alter hippocampal neurogenesis (ampicillin + sublactam, vancomycin, ciprofloxacin, imipenem plus cilastatin, metronidazole),^85^ anxiety and cognitive behaviors (ampicillin, vancomycin, metronidazole, ciproflaxin, neomycin or ampicillin, bacitracin, meropenem, imipenem, neomycin and vancomycin).^86^ Important side effects also include immune system perturbations.^1^ Studies focusing on the gut-brain axis can be compromised by the use of compounds able to cross the blood-brain barrier or those that are readily absorbed from the gastrointestinal tract. We know, for example, that metronidazole absorbed in the intestine can accumulate in the brain, where it may exert undesirable effects.^1^ If your study focuses on bacteriophage, it is important to consider phage-bacterial network interactions following antibiotic treatment.^87^

Three major considerations have to be taken into account regarding antibiotic-induced alterations in the gut: 1) depletion of the gut microbiota, 2) direct effects of antibiotics on host tissues and 3) effects of remaining antibiotic-resistant microbes. These alterations are dependent on the duration of the treatment and dosage of antibiotics and may lead to emergence of bacterial resistance. Indeed, bacteria compete with each other via a wide range of mechanisms, including the secretion of antibiotics.^88^ A disrupted healthy microbiota can lead to an expanded abundance of pathobionts – virulent species in the resident microbiota – resulting in aberrant pathobiont-induced innate immune signaling.^89^ To minimize bacterial resistance following an antibiotic treatment, high doses of antibiotics over a fairly short duration is preferable.^90^ This is consistent with studies suggesting that a short course of antibiotic treatment is sufficient to induce complete depletion without significant side effects.^37^ Antibiotics affect the microbiota of various host species in different ways.^37^ Functional studies on the gut microbiota that use antibiotic treatment must mention the limitations of this model and strive to control for the potential confounding effects as much as possible.

### Direct supplementation in conventionally colonized animals

Conventionally colonized animals are reared in an animal facility with their commensal microbiota. A few studies preferred not to use GF mice or a microbiota depletion-model and simply performed a fecal transfer in mice harboring their commensal microbiota.^91–93^ By reconstructing colonization patterns of human fecal microbes in mice with different genotypes (C57BL6/J vs. NSG) and with or without gut microbiota depletion using antibiotics, Zhou et al. found that mouse genotypes and native gut microbiota exert different selective pressures on exogenous colonizers, considered by the authors as colonization resistance.^91^ They concluded that depleting the mouse gut microbiota promotes colonization of human microbes.^91^ Conversely, another study looked at the results of transplantation in the same species (rat) when the recipient is treated with or without antibiotics. They concluded that antibiotic intake prior to transplantation did not increase the establishment of the donor phylotypes and that an indigenous gut microbiota can be reshaped without the use of antibiotics.^94^ To date, very few studies transferred microbiota into rodents simultaneously harboring their native commensal microbiota. In some cases, the use of conventional animals for FMT studies seems to be an adequate solution to limit the variables of the study. For instance, in the context of an infectious disease^95^ or after inducing a disease by injecting a chemical compound that mimics the symptoms.^5^ FMT in conventional animals can restore or help to improve the symptoms of a disease.

### Laxative-depletion

More recently, the use of laxatives as an alternative approach to counteract limitations of both gnotobiotic and antibiotic-treated rodents have been considered in FMT experiments. For example, it has been shown in conventional mice that the gut microbiota can be depleted by laxative exposure: 4 bowel cleansings with PolyEthylene Glycol (PEG) were used to empty the intestines and transiently decrease bacterial load by 90%.^96^ However, as there is little research using this method yet, it makes it difficult to compare across studies. More detailed studies on the influence of the PEG (or other possible laxatives) are necessary to allow us to discern the limitations of this model. However, three pre-treatment conditions (antibiotics, laxatives or no pre-treatment) have been recently compared.^97^ Interestingly, and perhaps not unexpectedly, antibiotics induced a more effective depletion of the intestinal bacterial community in the colon than induced by laxative treatment.^97^ However, disparities exists between the concentration of laxative (PEG) used in this versus other studies.^24^ It appears that laxative-induced depletion can be a valid method to induce gut microbiota depletion – however, at least 170 mg of PEG is needed to obtain a significant bacterial load reduction in adult mice.^96^ Moreover, its systemic effect or direct impact on the intestine is not yet well characterized. In humans, laxative depletion is the most popular method of depleting the microbiota in patients requiring FMT following *C. difficile* infection; however, it may simply reflect the common practice of bowel preparation prior to colonoscopy. At present, this method provides an alternative in newborns rodents or studies where antibiotic depletion or GF state is not suitable. However, it is unclear if this method can be successfully applied to all pathological conditions.

### Vertical microbiota transmission models

In 1995, Hirayama et al. demonstrated that GF mice, transplanted with microbiota from a human donor, could transmit the human gut microbiota to their offspring – thus circumventing the side effects of using a GF model by studying the next generation.^98^ Indeed, after conducting a long-term assessment of the transmission modes of bacterial genera – 11 generations – they showed that the majority of the murine gut microbiota was vertically inherited.^99^ More specifically, obligate anaerobes tended to be transmitted vertically whereas obligate aerobes tended to be transmitted horizontally. Since then, other studies demonstrated that bacteria were transferred to the offspring naturally from the mother and used it as a model of FMT in rodents.^100,101^ This model was later refined by spreading the corresponding FMT inocula on the abdominal and nipple regions several days after birth.^102^

Given that GF mice have many physiological, immune and neurodevelopmental alterations at baseline it makes it difficult to use them for certain causal inferences.^2^ Vertical transmission to the next generation can obviate these effects at least partially. In this regard, Sharon et al. revealed that colonizing GF mice with fecal microbiota from autistic human donors was sufficient to promote core behavioral symptoms in their offspring.^103^ The maternal gut environment during pregnancy is a contributor to metabolic programming of the offspring (i.e. can confer resistance to obesity).^104^ It is therefore plausible that administering short-chain fatty acid-producing bacteria in mothers might influence prenatal development of the metabolic and neural systems. Overall, this model allows a natural transmission of an “exogenous” microbiota without deficiency in development – as opposed to a GF development. In contrast, it has been shown in humans that viromes are unique to individuals regardless of their genetic relationship.^1^ However, the lack of information on the transmission of microbiota from the mother to the offspring prevents conclusions regarding the validity of this method for FMT studies.

### Bedding material, coprophagy and co-housing

Due to the coprophagic nature of rodents, some studies transfer the microbiota of donor rodents by transferring used bedding from the donor mouse to the cage of the recipient. This technique can also be used as booster inoculation, after gavaging rodents with FMT once, cages of recipient mice can be replenished with dirty bedding and fresh fecal pellets from donors several times a week.^105^ The recipient mice can also be recolonized by swabbing their mouths with homogenized donor fecal pellets prior to placing bedding and feces from them into the cages of the recipients.^54^

However, co-housing donor and recipient animals might lead to an untargeted and uncontrolled colonization. For example, results from a study where mice were caged together after receiving either a microbiota from a healthy or an underweight human donor, showed that colonization was mainly from mice receiving a healthy microbiota to mice with an undernourished microbiota but not the opposite.^106^ This suggests that it is challenging to control the outcome of a microbial transfer using this method of colonization. Moreover, it is possible that this method allows the transfer of aerobic and facultative anaerobic microorganisms but might not transfer strict anaerobes due to their exposure to an oxygenated environment. Currently, there are not enough studies comparing different methods of microbial transfer which prevents us from fully understanding the effectiveness of this method compared to the others.

### Comparisons of different models

#### The ideal donor

FMT treatments have become prominent tools in research due to their use in *Clostridioides difficile* infection, as antibiotic treatment induces a large disruption of the commensal gut microbiota community and functionality whereby the pathogen is then able to proliferate and cause disease.^107^ For this reason, a healthy and intact gut microbiota can outcompete and replace *C. difficile* in the intestinal habitat and this leads to effective treatment and resolution of the infection.^108^ The extent to which microbial engraftment is necessary compared to treatments that influence a shift in the microbiome of the recipient is likely dependent on the goal of the treatment. It appears that engraftment is less important if the purpose of the FMT is to treat infections such as *C. difficile*;^109^ whereas certain microbial taxa may need to be present from engraftment in order to introduce specific functional outputs (*eg. Ruminococcaceae, Verrucomicrobiaceae*, and *Lachnospiraceae)* in the case of cognitive improvement and reduction in inflammation following FMT in cirrhotic patients^110^- which is still to be fully established in FMT pre-clinical studies.

The goal of these studies is to better understand the influence of the gut microbiota in the desired model and to maximize the efficient use of animals, materials and labor. Herein we identify the route to optimally recapitulate the microbiota of the donor to recipient through FMT. These considerations are intended for both human and animal donors. It is necessary to characterize the donor in terms specific to the study as well as general health and lifestyle traits that may be pertinent. Reportable and easily obtained descriptors that have been identified to affect the gut microbiota should be collected when identifying donors: such as age, sex, diet, general health, medications, etc. Undoubtedly more information leads to more detailed selection criteria, however, donor information should be carefully considered and tailored to the goals of the study. Similarly, donor exclusion criteria include recent antibiotic use, illness, disorder, medication use, age and chronic disease. Additional factors should be considered dependent on the specific research goals of each study. It is best to analyze the gut microbiota of the donor before FMT to the recipient as this allows for the association of the donor with specific taxa and functional groups with the phenotype being studied. Then, individual donors with a combination of the highest phenotypic scores and selected gut microbiota profile traits can be selected for use. Furthermore, it is unknown to what extent the gut microbiota will be linked with different diseases and disorders, therefore, whether specific microbial groups are implicated in the causality or result as a downstream consequence of the phenotype will impact the results. It may be desirable to use phenotypic extremes of a population for the recapitulation of a donor profile in the recipient animal. However, in some cases, like in a highly heterogenous population, randomly chosen donors may function better. Patient donors commonly take medication for either the target phenotype of the study or other comorbidities; this should be accounted for in the study design. Ideally, fecal microbiota should be collected from donors without medication since some compounds are able to impact gut microbiota significantly,^111^ and therefore the medication remodeled gut microbiota can potentially be transferred to the recipient by FMT and exert downstream effects in the model. If donors without medication cannot be found – which is a difficult criterion to fulfill in some populations – then it is paramount to adequately control for the potentially confounding factors.

Microbiome composition, stability, and engraftment are determined by a number of factors^112^ such as genes, environment, diet and immune function that can impact how well the FMT is received. Donor microbiome composition and complexity (*i.e*. diversity) appear to be major determinants of transplant efficacy.^113^ Patients who have a successful clinical response to FMT (*i.e*. responders) usually exhibit a higher microbial diversity than those who do not (*ie*. non-responders).^114^ Conventional theory is that there are certain microbes fundamental to healthy functionality of the gut microbiota, which, through mutualistic mechanisms (like microbial cross feeding and pathogen exclusion), aid the colonization of other commensal microbes.^115^ However, proving this theory has proven to be an elusive and arduous task as there is tremendous inter- and intra-individual heterogeneity dependent on a multitude of different factors. One explanation is that the groups necessary to maintain a healthy microbiota are composed of guilds that provide specific functions^116^ within the gut while important clusters and specific members are commonly referred to as enterotypes and keystone species (e.g. *Bacteroidaceae, Bifidobacteriaceae, Lactobacillaceae, Eubacteriaceae, Prevotellaceae*).^117^ Furthermore, emerging evidence suggests that some FMT donors may result in higher efficacy to treat *C. difficile*, irritable bowel syndrome, and inflammatory bowel disease. These individuals are frequently described as super-donors.^118^ Common trends found in super donor FMTs are high microbial diversity, richness, and the presence of keystone species within the microbiota composition (e.g., butyrate producers within *Clostridium* clusters *IV* and *XIVa*).^113,119^ However, more research must be done to identify, evaluate, and utilize this concept to our advantage and to elucidate whether there are similar factors that influence cross-species FMT.

### The ideal recipient

#### Rodent recipient models

This review focuses only on rodent recipients as it is the most widely used model in pre-clinical research to assess causality of the intestinal microbiota. Rodents are commonly used as recipients of microbiota from human donors even though they might not be the most appropriate model for a successful engraftment given their disparities – compared to monkeys or pigs for example.^120^ Indeed, porcine models were used successfully but are not within the repertoire of most research groups in the field.^121^ On the same note, colonizing zebrafish with either zebrafish,^122^ mice^122^or human^123^ gut microbiota has also been the focus of FMT research. However, the transfer of behavioral phenotypes from human or rodent donors to rodent recipients has been demonstrated most frequently.^9,10^

When FMT is performed within the same species, there are fewer physiological dissimilarities within the GI tract (pH, morphology, diet etc.)^120^ and a greater likelihood that the microbiota of the recipient will resemble that of the donor. However, it seems likely that donor-specific taxa reliably colonize recipients only when rich donor material is transferred to mice originally colonized with a simpler microbiota.^124^ Several studies have compared humanized rodent models and concluded that rats outperformed mouse models – in terms of colonization efficiency – because they are more similar to humans and capture more human microbial species.^125^ Spatial organization of bacterial communities across and along the GI tract complicates microbiome analysis. The sample type used to extract bacterial genetic information is of crucial importance. Along the gastrointestinal tract, a gradient of pH, bile acid concentrations, oxygen levels and antibacterial products exists. Therefore, the microbial composition of feces and intestinal tissue is intrinsically different, because they are dictated by the radial gradient of oxygen and substrates provided by the host.^126^ Consequently, when a rodent is colonized by the microbiota originating from different gut regions of a donor – in this case a pig – different colonization patterns are obtained depending on the site of origin of the donor inoculum: jejunal, ileal, cecal, colonic, fecal or whole-intestinal microbiota.^127^ It was  demonstrated that the microbiota of a specific intestinal region selectively colonizes the corresponding intestinal region of the recipients; leaving the whole-intestinal microbiota inoculum as the most promising solution to reconstitute the entire microbiota of the whole gastrointestinal tract. For ethical and practical reasons, it may not always be feasible to collect tissue (biopsy) samples. In addition, fecal matter is usually collected to meet practical standards for the donors in terms of storage and shipment, and some investigators use samples that were collected anaerobically.

#### Sex differences

Despite sex being an important variable affecting the gut microbiota,^128^ the majority of scientific publications still present results from males only. It has been demonstrated that sex differences and hormonal effects on gut microbiota composition are important; by comparing 89 different inbred strains of mice – including C57BL/6 which exhibited high sex-specific differences and are widely used in FMT studies.^129^ Both sex and strains have a significant effect on the gut microbiota. To test the evolvement of sex hormones, they performed a gonadectomy in 3 different strains and were able to identify clear hormonal effects on gut microbiota composition. Markle and colleagues investigated how a male donor would influence a female recipient – with particular regard to age.^130^ Their data showed that in young female recipients, testosterone elevation was compatible with normal breeding behavior but not in adults; suggesting that if inter-sex transplantation needs to be done, puberty is probably a more appropriate period. Indeed, sex-specific differences in gut microbiota composition became evident at puberty and most apparent in adult mice. FMT to GF mice from same or opposite sex was done to investigate the stability of engraftment at 1 and 4 weeks following FMT administration; it appears that for both the microbiota first adapted to the sex of the recipient at week 1, then at week 4, gut microbiota composition was similar to the sex of the donor, regardless of the sex of the recipient.^131^ Therefore, sex-specific differences need to be considered while designing an FMT experiment to ensure valid conclusions for both genders: it is preferable that donors and recipients be of the same sex.^129^

#### Age of the recipient

The next important consideration is the age of the recipient rodents that allows the most efficient transfer of the donors’ gut microbiota. By transferring microbiota from a donor mice into juvenile or adult SPF mice, it was suggested that the engraftment was more efficient in juveniles rather than adults.^24^ This observation seems plausible given that the rodent microbiota is more stable at a later stage of life.^132^ With these collective results, they show that the use of juvenile mice subjected to initial microbiota depletion constitutes a valid alternative to GF mice in microbiota transfer studies.^24^ However, additional research should be conducted to understand the impact of age on microbiota transplant success. Indeed, age-related changes in the intestinal microbiota can have a significant impact on metabolism, immunity and behavior.^133^ If antibiotic-depletion is used during early life in a recipient animal, this can be associated with long-lasting metabolic consequences,^134^ even if the microbiota recovers following colonization. FMT experiments on rodents with a significant age difference influenced behavior,^135^ inflammation,^105^ neurogenesis,^136^ and intestinal morphology.^136^ When planning an experimental design with human donors, the ideal option would be to match the stage of life between human and rodent, or at least acknowledge the implications that the age of the model plays in the engraftment of the FMT and the measured outcomes.

#### Housing conditions

Important considerations such as housing conditions need to be discussed in an FMT experiment. First of all, rodents are coprophagic, therefore they cannot be caged together if they receive FMT from different donors unless this is an intentional experimental intervention. By comparing metagenome composition of mice depending on: strains from different suppliers, housing laboratory and low- or high- fat diets; it was shown that mouse provider and housing conditions had a pronounced effect on the composition of the gut microbiota.^137^ Although the mouse supplier is a factor that cannot be controlled when comparing between studies, housing condition is. On the other hand, when one becomes interested in human-to-mice FMT success with regard to housing conditions and mice coprophagy; it was found that in mice inoculated with the same donors and caged together in a very controlled environment – 2 mice per cage – the spread of microbes between cages occurs within each isolator.^91^ However, individually ventilated cages might be sufficient to prevent bacteria transfers between cages for at least 9 weeks when basic hygiene measures are applied when handling mice,^24^ but may compromise some behavioral readouts.^17^ Furthermore, cohabitation of mice with different phenotypes (e.g. lean vs. obese or aged vs young) can lead to important metabolic changes.^105^

These results imply that animals from the same experimental group must be in the same cage, as cross-contamination could occur and affect the results. To prevent coprophagy, collars can be used. However, coprophagy in rodents is beneficial for their metabolism; preventing it would affect a healthy energy balance, microbial diversity and downstream behaviors.^138^ When examining differences over broad range of animal facilities using standardized procedures while maintaining a constant host genetic background (C57BL/6 mice); increased variation was observed among mice held in open cages, in rooms where other strains are present, or in a less restrictive access policy.^139^ Furthermore, the gut microbiota of experimental animals is also influenced by contamination through shed skin or dust particles carried by other animals, caretakers, or scientists alike. The genus *Propionibacterium* is an indicator of human skin contamination of the mouse microbiota. Despite all efforts to maintain hygiene and standardize conditions and procedure, each facility, and even each room in a facility, harbors its own unique combination of a multitude of variable factors which will give rise to distinct microbiota configurations. The impact of these differences is an important but often unknown factor in FMT studies.

#### Diet

Diet has a great influence on intestinal microbes,^112^ and therefore demands particular attention for studies involving changes in gut microbial population. Many studies have demonstrated that FMT procedures from an animal or human with a specific diet to another animal can dramatically impact host physiology and metabolism; and thereby be an important confounding factor if not considered properly. Therefore, it is important to consider how the diet of the donor may affect the recipient^140^ but also the extent to which the diet of the recipient may affect the outcome of the microbial transfer.^141^ This was demonstrated elegantly in 2013, by transplanting the microbiota of human twin pairs discordant for kwashiorkor – a severe form of protein malnutrition – into GF mice. As expected, transplantation of the microbiota of the kwashiorkor co-twins in mice resulted in a transfer of phenotype compared to mice harboring the healthy microbiota of the sibling. Surprisingly, the weight loss phenotype was not the only the result of the microbiota transplant, but rather depended on the combination of the diet of the recipient (chow, low protein or high protein diet) and the transplanted microbiota. If the diet switched from low-protein to high-protein in recipient animals, all animals rapidly gain weight again.^140^ The fact that diet had such a significant impact in this study compared to fecal transplantation can also be explained by the fact that there was only one gavage for fecal transplantation followed by 63 days of different diet.

These results indicate that the microbiota of donors and the diet of both the donor and the recipient has a huge impact on the transfer of phenotype in gnotobiotic animals. Turnbaugh et al. also demonstrated that colonization of human or murine microbiota in GF mice is greatly influenced by diet: they demonstrated that a switch of diet – a low-fat, plant polysaccharide-rich or a high-fat, high sugar “western” diet – was able to shift the structure of the microbiota within a single day and altered microbiome gene expression of the mice.^100^ Moreover, if human donors are used, inter-species feeding differences should be considered as it shapes the gut microbiota differently.^142^ For example, when assessing the stability of human fecal transplantation in GF mice with a “humanized” diet – as opposed to mice on a regular chow diet: it was found that a humanized diet allowed a better retention of human gut microbiota in recipient mice^101^ and a decrease in enterotype changes.^143^ For gnotobiotic rodents, the diet can be treated: autoclaved or irradiated. It is important to know that these treatments influence the quality of the diet and no longer contain all the nutrients necessary for the development of a healthy microbiota.^139^ In such cases, a laboratory autoclavable rodent diet can be used, as it is supplemented with higher levels of nutrients to compensate losses during autoclaving. We might underestimate the impact of diet when conducting an FMT study, which is especially important for human-to-rodent FMT. It must be kept in mind that humans and rodents have very distinct diets, in addition to having many physiological differences, making it a challenge to obtain a humanized-microbiota model in rodents with a high percentage of microbial transmission.

#### Host and genetics

The current literature lacks information about which strain of rodents are best suitable for fecal microbiota transplant whilst genetics influence gut microbiota composition in widely used laboratory mouse strains.^144^ Table 7 summarizes which strains of rodents have been used over the past 30 years according to the topic of the study. If some information are needed to estimate the microbial composition of a specific strain of mice in healthy condition, a database has been compiled called “Murine Microbiome Database” with 9 common strains of laboratory mice.^458^ It was demonstrated that colonization patterns differed between mouse strains by comparing 23 different strains of gnotobiotic mice receiving the ASF gut microbiota.^459^ This illustrates how a transplant of only 8 bacterial strains can vary depending on host genetics. Korach-Rechtman et al.^460^ went even further by crossbreeding two strains of mice BALB/c and C57BL/6 J and studying the F_1_ offspring derived from their reciprocal crossbreeding (♀C57BL/6 J × ♂BALB/c; ♀BALB/c × ♂C57BL/6 J). They demonstrated that twelve taxa were shown to have genetically controlled gut persistence. This is in agreement with previous studies showing that some bacterial phylotypes appear to be discriminative and strain-specific to each mouse line used.^461^ While the two genetically distinct parental inbred lines presented important microbiota differences, their hybrids offspring presented a very similar microbiota; highlighting the importance of genetic effect on microbiota composition. Finally, they analyzed to what extent the inherited microbiota would be disrupted by co-habitation with one of the parental strains, allowing bacterial transfer by co-habitation and coprophagy. Interestingly, some taxa were modified by cohabitation but returned to their inherited microbiota composition after separation. This suggests that for a microbial phenotype of the donor to be sustainable over time, the microbiota of the donor must be inoculated repeatedly throughout the whole duration of the FMT experiment (e.g.: co-habitation, several inoculations across time).

**Table 7.** Rodent strains used in preclinical FMT studies over the last 30 years in the most prevalent topics

DISEASES	DONORS	RECIPITENTS	REFERENCES
Aging	Mice	Mice: C57BL/6	^32,43,105,136,145,146^
		Mice: C57BL/6 J	^147^
		Mice: C57BL/6N	^62^
		Mice: BALB/c	^105,148^
		Mice: Swiss Webster	^149^
		Mice: SAMP8	^150^
		Mice/C3H/HeN	^151^
	Rats	Rats: Dahl	^152^
		Rats: Sprague-Dawley	^153^
	Human	Mice: C57BL/6	^154^
		Mice: C57BL/6 J	^155,156^
Alcohol-related disorders	Mice	Mice: C57BL/6	^157,158^
		Mice: C57Bl/6 J	^159,160^
	Rats	Rats: Sprague Dawley	^161^
	Human donors	Mice: C57BL/6 J	^162,163^
		Mice: NSG	^164^
Alzheimer’s disease	Mice	Mice: C57BL/6	^43^
		Mice: ADLPAPT	^165^
		Mice: SAMP8 mice	^150^
		Mice: APPswe/PS1dE9 transgenic (Tg) mouse model	^7^
	Rats	Rats: Sprague Dawley	^166^
	Human	Mice: C57BL/6N	^167^
Autism-spectrum disorder	Mice	Mice: C57BL/6 J	^168,169^
	Human	Mice: C57BL/6 J	^103^
		Mice: C57BL/6N	^170^
Cancer	Mice	Mice: C57BL/6	^171–182^
		Mice: C57BL/6 J	^35,183–186^
		Mice: ICR	^187^
		Mice: Swiss webster	^173,174,188^
		Mice: BALB/c	^171,189–192^
	Rats	Rats: Sprague-Dawley	^193,194^
	Human	Mice: C57BL/6	^71,195–197^
		Mice: C57BL/6 J	^198^
		Mice: C57BL/6, ALB/c or IQI	^199^
		Mice: 5TGM1	^200^
		Mice/C3H/HeN	^201^
Cognition	Mice	Mice: C57BL/6	^43,145,202^
	Rats	Rats: Sprague-Dawley	^153^
Colitis	Mice	Mice: C57BL/6	^74,178,179,203–236^
		Mice: C57BL/6 j	^95,237–247^
		Mice: C57BL/6NTac	^248^
		Mice: C57BL/6 NCr	^249^
		Mice: Swiss Webster	^229,250–252^
		Mice: BALB/c	^191,239,253–261^
		Mice: CBA/CaJ	^230^
		Mice: 129SvEv	^262^
		Mice: CBA and Swiss Jim Lambert (SJL)	^263^
	Rats	Rats: Sprague-Dawley	^264–267^
		Mice: BALB/c	^261^
	Human	Mice: C57BL/6	^268^
		Mice: C57BL/6 J	^268–270^
		Mice and Rats: BALB/c and Sprague-Dawley	^271^
*Clostridium Difficile* infection	Mice	Mice: C57BL/6	^272^
		Mice: Swiss-Webster	^273^
	Human	Mice: C57BL/6	^274–276^
		Mice: C57BL/6 J	^277,278^
Diabetes	Mice	Mice: C57BL/6	^279^
		Mice: C57BL/6 J	^279^
		Mice: C57BL/6 NTac	^280^
		Mice: db/dd and C57BL/Ks	^281^
		Mice: Kunming	^282^
		Mice: NOD	^283–285^
		Mice: NOR mice	^285^
		Mice: BALB/c	^286^
	Rats	Rats: Sprague Dawley	^287^
	Human	Mice: C57BL/6 J	^288^
		Mice: NOD	^289^
		Mice: db/db and db/m unknown genetic background	^290^
Depression	Mice	Mice: C57BL/6	^61,291,292^
		Mice: C57BL/6 J	^293^
		Mice: BALB/c	^294^
	Rats	Rats: Sprague Dawley	^10,295,296^
		Rats: Long–Evans	^297^
		Rats: Flinders sensitive line and Flinders resistant line	^298^
		Rats Lewis	^299^
		Rats: Wistar	^300^
		Mice: C57BL/6	^46^
	Human	Mice: Kunming	^301,302^
Exercise	Mice	Mice: C57BL/6 J	^303^
		Mice: C57BL/6 JNarl	^33^
Irritable Bowel Syndrome/Irritable Bowel Disease	Mice	Mice: C57BL/6	^174,251,304^
		Mice: Swiss Webster	^174,251^
	Rats	Rats: Sprague-Dawley	^305^
		Rats: Long-Evans	^306^
		Rats: Wistar	^307^
	Human donors	Mice: C57BL/6	^308–311^
		Mice: ATG16L1T300A KO mice unknown genetic background	^312^
		Rats: Sprague-Dawley	^313^
		Rats: Fisher 344 albinos	^314^
Liver-associated conditions	Mice	Mice: C57BL/6	^315–321^
		Mice: C57BL/6 J	^322–327^
		Mice: DBA/2 J	^327^
		Mice: IRC	^328^
		Mice: Swiss Webster	^329^
	Rats	Mice: C57BL/6	^330^
		Rats: Sprague-Dawley	^331^
	Human	Mice: C57BL/6	^38^
		Mice: C57BL/6 J	^96,163,332,333^
		Mice: SAMP	^334^
		Mice: Swiss/NIH	^335^
		Rats: Sprague Dawley	^336^
		Rats: F344	^337^
Malnutrition	Mice	Mice: C57BL/6	^338^
		Mice: A/J and C57BL/6 J	^339^
	Human	Mice: C57BL/6 J	^106,340,341^
Metabolic syndrome	Mice	Mice: C57BL/6	^342–345^
		Mice: C57BL/6 J	^346,347^
		Mice: C57BL/6 N	^348^
		Mice: ICR	^347,349,350^
		Mice: Swiss Webster	^251,351,352^
		Mice: BALB/c	^353^
	Human donors	Mice: Swiss-Webster	^352^
Multiple sclerosis	Mice	Mice: C57BL/6	^354,355^
		Mice: C57BL/6 J	^356,357^
	Rats	Rats: Dark Agouti	^358^
	Human	Mice: C57BL/6	^359^
Nonalcoholic Fatty Liver Disease (NAFLD)	Mice	Mice: C57BL/6	^360,361^
		Mice: C57BL/6 J	^362^
Nonalcoholic steatohepatitis (NASH)	Mice	Mice: C57BL/6	^363^
Obesity	Mice	Mice: C57BL/6	^75,141,222,364–373^
		Mice: C57BL/6 J	^346,374–391^
		C57Bl/6 N	^392–395^
		Mice: C57BL/6 NTac	^396^
		Mice: C57BL/6 JNarl	^33^
		Mice: SwissWebster	^149,397^
		Mice: ICR	^398^
		Mice: ob/ob	^399^
		Mice: Atg7	^400^
	Rats	Rats: Sprague Dawley	^401–406^
		Rats: Wistar	^45^
		Rats: LZ and ZDF	^407^
	Pigs	Mice: C57BL/6 J	^408^
	Human	Mice: C57BL/6	^409,410^
		Mice: C57BL/6 J	^411–415^
		Mice: Swiss Webster	^416,417^
		Mice: db/db and db/m unknown genetic background	^290^
Pancreatitis	Mice	Mice: C57BL/6	^418–420^
		Mice: C57BL/6 J	^421,422^
		Mice: MRL/MpJ	^423^
		Mice: NOD/MrkTac	^424^
Parkinson	Mice	Mice: C57BL/6	^5^
		Mice: C57BL/6 J	^425^
Polycystic ovary syndrome	Mice	Mice: C57BL/6 J	^426^
	Rats	Rats: Sprague-Dawley	^427^
	Human	Mice: C57BL/6	^428^
Schizophrenia	Human donors	Mice: C57BL/6 J	^429,430^
		Mice: Kunming	^431^
Sepsis	Mice	Mice: C57BL/6	^432–435^
		Mice: C57BL/6 J	^436^
		Mice: Swiss Webster	^435^
	Rats	Rats: Sprague-Dawley	^437,438^
		Rats: Wistar	^439,440^
	Human	Mice: C57BL/6	^66^
Stress	Mice	Mice: C57BL/6	^441^
		Mice: C57BL/6 J	^72,293,442,443^
		Mice: SKG and BALB/c	^444^
		Mice: BALB/c	^14^
	Rats	Rats: Wistar-Kyoto and Spontaneously Hypertensive Rats	^445^
		Rats: Sprague-Dawley	^446–448^
	Human	Mice: C57BL6/J and NSG	^449^
Stroke	Mice	Mice: C57BL/6	^32,36,146,450^
		Mice: C57BL/6 J	^451^
		Mice: BALB/c	^452^
	Rats	Rats: Sprague Dawley	^453,454^
		Rats: Dahl	^455^
	Human	Mice: C57BL/6	^456,457^

To conclude, this study shows that some taxa might be present in the offspring independently of genetic transmission; but also, that relationships of genetic dominance and recessivity exists which influence the microbial composition of the offspring. This has been exemplified in a study showing that administration of *Lactobacillus johnsonii* level decreased rapidly after oral administration in BALB/c mice but not C57BL/6 J mice.^462^ Interestingly, inter-strain colonization of BALB/c and NIH Swiss mice into GF mice was able to alter behavioral phenotype to resemble the behavior corresponding to the microbiota of the donor.^463^Although FMT between different mouse strains may result in similar intestinal microorganisms between donor and recipient,^339,464^ transplantation is optimal if the mouse strains are similar since the host will not exert as much selective pressure on the microbiome of the donor.^465^

Lastly, the use of knock-out rodents has been coupled with FMT experiments. Interestingly, deletion of a single gene can cause substantial alterations in the microbiota^466^ and thereby be an important confounding factor. In some cases, FMT can reduce the severity of a phenotype in WT animals. For example, in experimental necrotizing enterocolitis, which was reduced in severity in WT but not Grx1-/- mice.^203^ This suggests that an important gene can modulate the benefits of FMT, opening up the possibility of specifically targeting a mechanism of interest. Therefore, such study design requires appropriate control groups or preliminary data comparing the composition of the gut microbiome of the wild-type and knock-out mouse models. Further information is required regarding how different strains of rodents differ in terms of microbial composition and how it can influence the outcomes of FMT protocols.

#### FMT induced microbial, metabolic and immune changes in the host

The genetic background and microbial profile of the host plays a fundamental role in associated microbial, metabolic and immune changes following FMT. Indeed, FMT experiments not only induce changes in the microbial profile of the host, but also causes important metabolic and immune changes. This was explored more extensively in clinical studies where it was shown, for example, that the metabolic benefits associated with a transfer of intestinal microbiota from a lean individual to an obese patient with metabolic syndrome is driven by baseline microbial composition of the host.^467^ However, it is unclear how the colonization of a GF animal following different FMT preparations will affect its immune system and intestinal morphology. Indeed, the intestine comprises multiple mechanisms to ensure a good balance between the preservation of the bacterial members of the microbiota and the elimination of pathogens. Distinct sites in the gastrointestinal tract are composed of different cell types (including Paneth and goblet cells) and mechanisms of action (mucus secretion, immune activation) that act together to preserve location-specific intestinal homeostasis.

To elucidate the immune mechanisms involved in colonization, some researchers have analyzed innate and adaptive immune responses following colonization in GF mice.^468^ To start with, they observed a time- and region-dependent enrichment of genes involved in innate and adaptive immune responses – mainly involving T cells – with the largest proportion of differentially expressed genes being involved in the development of the mucosal immune system. They concluded that a novel state of homeostasis was achieved 30 days post-colonization in a region-dependent manner: homeostasis appeared to be established after 8 to 16 days in the colon, whereas in the jejunum and ileum 16 to 30 days were required.^468^ Importantly, 4 days post-colonization represented an important turning point: strong induction of innate immune functions followed by the stimulation of adaptive immune response, secretion of antimicrobial peptides by Paneth cells and biochemical changes in the mucosal barrier. At the anatomical level, FMT reduced cecum weight considerably, enhanced crypt depths in the whole intestine and increased connective tissue cells in the lamina propria in both the jejunum and the ileum.^468^ However, one study investigated bacterial colonization of GF mice depending on whether the inoculum of the donor (pig) was provided from the jejunum, the ileum, the cecum, the colon, feces or whole intestine.^127^ They found that bacterial colonization across different gut segments resulted in anatomical differences in the gut following FMT of the recipient mouse. A similar study with a mouse-to-mouse FMT would be interesting to discern inter- and intra-species FMT differences in FMT grafting according to the origin of the inoculum.

Regarding the impact of colonization on metabolic changes, it appears that the introduction of an exogenous microbiota into a GF mouse greatly impacts host fat storage by affecting hepatic lipogenesis and adipocytes leading to an increase in body fat content despite a reduction in food consumption.^469^ These are critical points of information when phenotypic, metabolic or behavioral changes are observed in studies on FMT, as the involvement of the intestinal microbiota can be direct – through secreted or metabolized molecules – or indirect by modifying the immune and physiological environment of the intestine, which has repercussions on the extra-intestinal organs. Moreover, it seems like colonization is time- and region- specific^468^ which implies that depending on the aim of the study, one should wait from 1 to 4 weeks for the newly established gut microbiota to be stable but also for its environment to achieve a new state of homeostasis.

### Fecal microbiota transplant preparation

**Figure 3.**
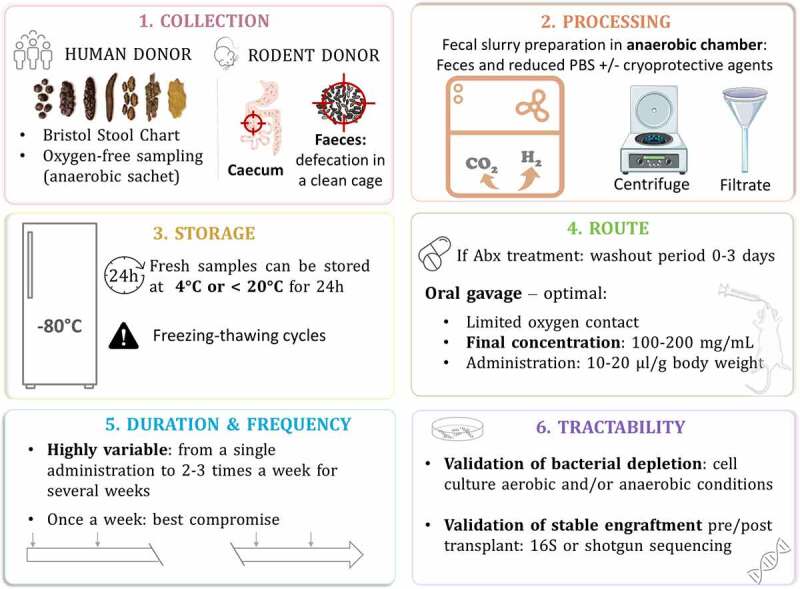
Fecal microbiota transfer: from donor sample collection to the tractability of the microbial transplant

To enhance comparability in the human microbiome field, it is necessary to coordinate and provide standard operating procedures. Some researchers in the field have greatly contributed by launching the International Human Microbiome Standards project where an entire section is dedicated to human sample collection and processing standards and provides procedures for different conditions (http://www.human-microbiome.org/). Regarding FMT from rodent donors, there is currently no equivalent level of standardization and guidance on minimum requirements. Establishing standardized methodologies is critical, with many studies indicating that techniques for processing stool samples vary depending on the experimental design, feasibility and facilities. Figure 3 above summarizes key steps for FMT from donor sample collection to the tractability of the microbial transplant.

#### Collection

Microorganisms in the colon are mostly strict anaerobes and oxygen is detrimental to their survival. Collection steps need to take this into consideration. If the donor is human, the feces can be contained in the sample box with an anaerobic sachet that will keep the sample in an oxygen-free environment. The Bristol Stool Chart can be used to record the appearance of collected material, with scaling ranging from 1 (constipation) to 7 (diarrhea).^470^ International Human Microbiome Standards project offers some guidelines for collection of human samples under different circumstances.^471^ Ideally, to maximize the preservation of microorganisms, all fecal samples should be kept at 4°C or on ice after collection and during transportation (but should not be frozen as freeze-thaw cycle are damaging for microorganisms); then extracted ideally within 1 hour of collection or a maximum of 24 hours.^472^ However, some logistical constraints prevent researchers from doing this. Successful transfer of an autistic phenotype by FMT was possible even when the sample was frozen directly at −80°C and processed just prior to transplantation.^103^

Regarding the collection of mouse feces, donor mice can be placed in a clean cage and allowed to defecate normally. The amount of feces needed for the inoculum preparation would depend on the experimental design of the study. Researchers can then use individual sterile tools to collect and place fecal pellets in a sterile cryo-vial prior to processing in an anaerobic chamber.

#### Processing

Several studies compared the quality of donor samples when used fresh or frozen for FMT studies. Here again, the methods used to determine the quality of the samples differ between studies and therefore leads to different conclusions. One study found no significant differences in terms of richness, diversity and community structure in mice receiving FMT prepared from frozen donor feces; however, frozen samples were stored for a short time at −80°C.^124^ Another study found no differences in aerobic and anaerobic populations usinfigug culture-based analysis between the fresh, snap or −80°C frozen samples. However, they treated all samples with a maximum recovery diluent used to support a maximal recovery of microorganisms. This suggests that frozen samples can constitute a good alternative to the use of fresh sample, but with addition of protective solution prior to freezing.

Unlike previous publications, Papanicolas et al.^473^ showed a significant effect of freeze-thaw cycles which reduced viability to 23% – despite the use of a cryoprotective agent (glycerol) without significantly affecting taxa richness in the sample. Interestingly, they observed an important inter-donor variation in the impact of sample processing. Indeed, microbiota composition can vary from one donor to another in terms of richness of microorganisms vulnerable to oxygen exposure and freezing. Therefore, if the goal of the study is to transfer viable bacteria, we recommend processing the fecal sample in an anaerobic chamber and with the use of a cryoprotective agent if a freezing step is required to enhance bacterial survival. Even when processed in anaerobic conditions, a considerable proportion of bacteria will be damaged or deceased.^473^ First, the stool sample needs to be homogenized manually to avoid analytical biases due to the heterogeneity of fecal samples. Then, the fecal slurry can be homogenized in autoclaved reduced PBS and glycerol supplemented with L-cysteine hydrochloride, filtered and/or centrifuged.^474^ The addition of cryoprotective agents such as glycerol, skim milk, maltodextrin, yeast extract and antioxidants – like sodium ascorbate or cysteine – can help reduce damaging effects caused by stresses due to freezing affecting both physical and biological bacteria properties.^475^ Regarding fecal slurry preparation with rodent donors, most studies processed samples by diluting it with autoclaved, filtered water and homogenized the whole preparation using a tissue lyzer. Homogenates should then be passed through a 30–70 µm pore-size nylon filter to remove large particulate and fibrous matter to generate fresh fecal slurries.

#### Storage

For human samples, if time before processing to transplantation exceeds 24 hours, fresh samples should not be exposed to temperatures above 20°C, and refrigeration at 4°C can be a safe option.^476^ Importantly, the freeze-thaw cycles are more deleterious to bacteria than duration of cryoconservation.^473^ For preservation of bacterial community structure, fecal samples should be frozen within 2 days of collection and up to 2 years at −80°C which leads to minimal changes in the microbial community.^477^ By analyzing DNA of fecal samples after storage at −80°C for 14 years, it was concluded that microbial profiles are preserved and robust to this extended storage period.^478^

#### Route

When rodents are treated with antibiotics, generally a washout period of up to 72 hours is allowed without antibiotic consumption prior to the first FMT administration. The majority of FMT studies in rodents introduce the fecal material by oral gavage: a dose-controlled and efficient option to administer fecal microbes without damaging anaerobes because the contact with oxygen is limited. The volume generally administered in rodents is 200 μL at a final concentration ranging from 100 to 200 mg/mL.

#### Duration and frequency

These are the most variable parameter across studies. The frequency and duration of FMT administration range from a single administration to twice or three times a week for several weeks. To date, there is a lack of studies comparing transplantation according to the frequency of FMT administration. It has been suggested that repeated gavage at very short intervals (i.e. daily) disturbs the newly established ecosystem and that FMT once a week might be a good compromise.^96^ Importantly, several studies highlighted that repeated gavage instead of a single gavage increases similarity to donor microbiota.^11,96^ A stable microbial graft can be achieved only when the whole intestine has reached a new state of homeostasis with its new bacterial population. A few studies demonstrated that it can be achieved in 28–30 days after the first inoculation.^11,468^ Interestingly, this seems region-dependent where it takes 8–16 days in the colon and 16–30 days in the small intestine.^468^

#### Tractability

Validation of bacterial load is advisable to establish the extent to which depletion is achieved and maintained. It can be done before and after antibiotic treatment, but also every week after the first transplant to see how the exogenous microbiota is being engrafted. Culture-based methods are useful to assess the colony-forming units (CFUs) from fecal samples plated in aerobic and/or anaerobic conditions on nonselective media. In most cases, quantitative PCR of the gene encoding 16S rRNA is used as it allows for culture-independent assessment of gastrointestinal bacterial load.

By using different sub-regions of the 16S rRNA gene, one study showed that sequencing full length of 16S rRNA provides a real and significant advantage over sequencing commonly targeted variable regions to provide taxonomic resolution at species and strain level.^479^ Shotgun metagenomics is an exhaustive method to quantify microbial populations and to assess functionality. It is important to monitor the establishment of the engrafted microbiota, in order to be able to assess the extent to which an exogenous microbiota can be transferred.

### Emphasis on the experimental design

**Figure 4.**
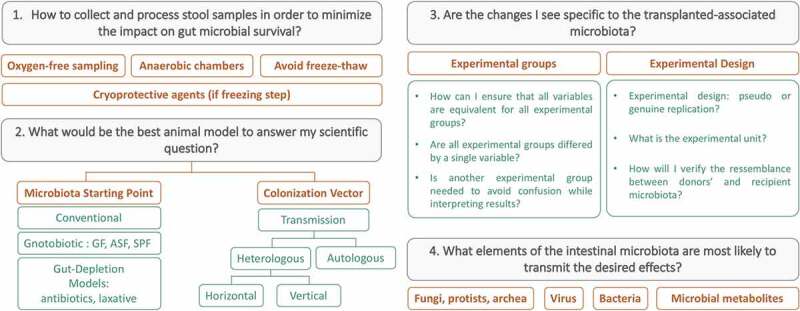
Key questions when designing an FMT experiment

Each step is critical when designing an FMT study as they all significantly influence the final outcome and data generated. Figure 4 provides key questions to be answered at the early stage of the experimental design.

To begin with, consideration should be given on how to collect and process donor stool samples in order to minimize its impact on microbial survival in the gut. Secondly, deciding on which animal model to use as a recipient of FMT should depend on its limitations and whether it fits the goals of the study. The initial starting point of the microbiota was developed extensively in the first parts of the review. However, several possibilities for transmission exists: it can be autologous (microbial transfer from the same animal) or heterologous (microbial transfer from a donor distinct from the recipient). If heterologous, it can be done vertically – from a parent to an offspring – or horizontally.

To be sure that the changes one sees are specific to the transplanted-associated microbiota, each experimental group should differ by only one variable between them, which is not always the case in FMT studies. The common independent variable in FMT experiments is the intestinal microbiota: the goal is to compare two experimental groups that do not have the same intestinal microbiota in order to compare the dependent variable(s) of interest (e.g. behavioral tests, molecular measurements etc.). Therefore, it is important that all the experimental group are treated similarly (e.g.: antibiotic treatment, laxative depletion, diet, housing conditions etc.) but differs only by the FMT treatment (control or disease of interest).

The same is true for GF animals that one wishes to recolonize with a microbiota of interest. A control group comprising only conventional animals will be insufficient to determine whether the differences are due to the GF status of the animal or as a result of its newly established microbiota. (see section: Gnotobiotic Animals)

Combination of two or more animal models as recipients of FMT (GF, gut microbiota-depleted, transmission via F1 generation etc.) would allow the study to be more robust if we see the effect of interest in two different animal models. GF studies could also be supplemented with microbiota-depleted animal experiments to highlight a specific time-window where the effect is expected. To better understand a mechanism of interest, it is possible to recolonize GF animals with a specific consortium of microbes tosee whether they are responsible for the effect observedafter a full FMT.

In addition, FMT experiments can lead to a wide range of experimental designs: some studies are designed to have one recipient per donor, whereas other experimental designs pool fecal samples from several donors for FMT into one recipient animal. To account for experimental and individual variability, you can also have several recipients per donor or per pooled samples. With such variability in experimental designs, it is becoming more difficult to compare across studies and to determine which method is the most appropriate. Walter et al. highlighted an important bias of experimental designs in FMT studies, that has a huge impact on statistical analysis and thereby, the conclusions it led to.^480^ Indeed, in FMT studies, sample size calculation can be challenging, and the experimental unit may not necessarily necessarily equal the number of recipient animals. More commonly, the sample size of the study corresponds to the number of donors per animal or group of animals if the samples are not pooled together.^480^ Therefore, if one donor is used to gavage several mice, the sample size would be ‘N = 1' as the group of mice receiving FMT from the same donor will be technical rather than biological replicates. If the donor samples are pooled before inoculation in rodents, it would limit the potential understanding understanding of inter-individual variability of the donors. Indeed, pooled FMTs do not resemble individual donors, but rather a combination of multiple donors, which might not lead to an accurate animal model for representation.

Importantly, it emphasized that the transfer of different gut microbial populations via FMT does not necessarily produce the same output in recipient mice, since the gut microbial profile of the recipient plays an important role in FMT engraftment – suggesting that technical replicates for each donor are necessary.^124^ Where possible, researchers should monitor the successful engraftment of the transplanted donor microbiota to the recipient animal (preferably at species level), and not solely focus on whether phenotypes of interest were successfully transferred, as we do not know the extent to which the transplanted microbiome must be engrafted in order to achieve a significant phenotypic or molecular change. 16S or shotgun sequencing methods require DNA extraction from feces samples to analyze gut bacterial composition. If using a microbiota-depletion model (e.g. laxative or antibiotic treatment), depletion should be monitored at different stages either by cell culture or 16S sequencing.

Once again, FMT studies present a disparity in DNA extraction methods – with some methods performing better than others. By carrying out replicate DNA extractions (with different researchers, different reagents, different protocols), it was concluded that it contributes negligibly and still leads to consistent results.^477^ DNA extraction kits can be chosen accordingly to the purpose of study, but it is preferable to include a repeated bead-beating step and a heating step for sample lysis and controls (internal and external) must be included.^481^ Verification of DNA quality and quantity from fecal samples is highly recommended before sequencing as it will directly affect downstream results.

If the desired effects are transmitted, the next question would be to understand what elements of the FMT preparation is most likely to transmit the desired effects as the preparation will include: bacteria, viruses, fungi, protists, archaea, microbial components and metabolites. Indeed, there is still much to be discovered about the viable and active components within the transplant.^473^ The fecal inocula used for FMT are typically filtered or centrifuged prior to transplantation. Many active components are remaining within the fecal filtrate, including small molecules, metabolites, nucleic acids, and viruses that are capable of freely passing through the typical filter pore size (30–70 µm). It is possible to use size stratification filtration techniques combined with other methods to target specific molecules or viruses.^482^ The effects of more intensive filtering and transplantation of only fecal metabolites has not yet been tested. It is important to note that these biologically active small compounds and viruses are also components, at least initially, in FMTs. It remains unclear if they persist for a sufficient period of time or at the concentrations necessary to impact experimental readouts. The most predominant viral entity in the gastrointestinal tract and feces is the bacteriophage (or simply phage), which infect and multiply within bacteria.^483^ Early evidence indicates that fecal virome transplants are capable of changing the gut microbiota of the recipient and impacting host health.^484^ This calls into question whether the host is responding to the microbiota, directly, to the virome transplant or a combination of both. Unraveling this interaction is another avenue for future research and will likely offer more treatment options.

More rigorous and critical approach for inferring causality in the microbiome field is needed along with recommendations that might help researchers with planning a correct experimental design and appropriate statistical approach for future FMT study.^480^ Future efforts should also focus on implementing minimum reporting requirements for preclinical FMT methodology to improve reproducibility and consistency across the field.

## Conclusion

FMT is a powerful tool to understand the involvement of gut microorganisms in health and diseases. Today, we can study the impact of the gut microbes without germ-free facilities, however we must rely on alternatives such as antibiotic or laxative treatments to deplete gut bacteria prior to FMT. These methods have been helpful to characterize the involvement of microorganisms in numerous diseases; however, it should be coupled with targeted mechanistic studies to fully understand the impact of these microbes and to what extent they are implicated in the phenotype of interest. To understand fully the impact of the intestinal microbiota in health and disease, we need to deepen our knowledge and find new methods to investigate the mechanisms of action of the relevant microorganisms.

In the meantime, experimental protocols require better design to counter the biases associated with this method and to establish rules of standardization, especially in the case of human-microbiota associated rodents. To ensure maximum bacterial survival, we recommend that sample collection and processing – when possible – should use anaerobic conditions at both stages of collection and processing,^473^ even though there is evidence of achieved phenotype transfers without. Furthermore, it is important to acknowledge the current gaps and limitations of metagenomic sequencing to be able to interpret published data with caution.^485^ Biases in microbiome studies can be introduced at every step: from sample collection to data analysis – all of which can impact interpretation and discovery.

Recently, a mathematical model was proposed to quantify bias, to partition bias into steps such as DNA extraction and PCR amplification, and to reason through the effects of bias on downstream statistical analyses.^486^ Future research is needed to improve this technique and to understand the best experimental approach one can use to answer a specific question and understand: how genetics and sex of the rodents influences the transplantation, gut microbiota depletion agents (side effects, concentrations to be used, frequency of administration, length of treatment) and their impacts on fecal transplantation, what components of the fecal or cecal microbiota are having the desired effect in an FMT and limitations of sequencing and culturing techniques.

In conclusion, it is imperative to maximize the standardization of FMT studies – from experimental design to statistical analysis – to be able to compare different studies and to discern whether changes observed are the cause, the consequence or indirectly related to the area of study in question. FMT is a good model to assess causality but we need a more complete understanding of specific mechanisms involved. This understanding will enable us to translate this research for the benefit of human conditions and diseases, by providing microbiota-based treatments  with the help of defined consortia or with specific strains administered in probiotics or by diet.
